# Supplementary material for: A quantitative and spatial analysis of cell cycle regulators during the fission yeast cycle
Source: Proc Natl Acad Sci U S A. 2022 Aug 29;119(36):e2206172119. doi: 10.1073/pnas.2206172119 (PMC9457408; doi:10.1073/pnas.2206172119)
Supplement: Supplementary File [file pnas.2206172119.sapp.pdf]

PNAS

Supporting Information for

A quantitative and spatial analysis of cell cycle regulators during the fission yeast cell cycle

Scott Curran, Gautam Dey, Paul Rees & Paul Nurse

Corresponding author: Paul Nurse
Email: paul.nurse@crick.ac.uk

This PDF file includes:

Figures S1 to S3
Tables S1 to S2

Fig. S1.

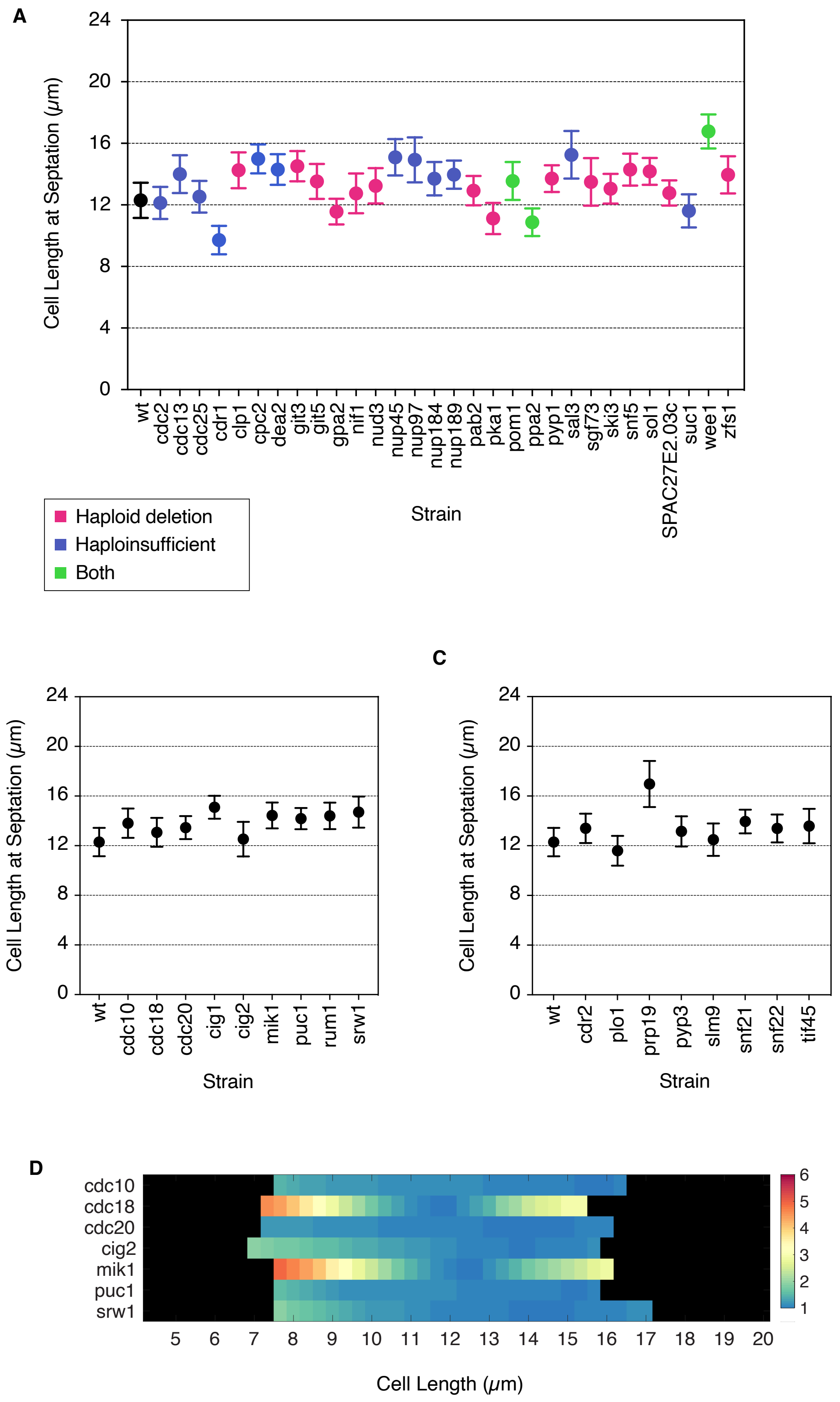
A
Cell Length at Septation (μm)
0
4
8
12
16
20
24
wt
cdc2
cdc13
cdc25
cdr1
clp1
cpc2
dea2
git3
git5
gpa2
nif1
nud3
nup45
nup97
nup184
nup189
pab2
pka1
pom1
ppa2
pyp1
sal3
sgf73
ski3
snf5
sol1
SPAC27E2.03c
suc1
wee1
zfs1
Strain
Haploid deletion
Haploinsufficient
Both
B
Cell Length at Septation (μm)
wt
cdc10
cdc18
cdc20
cig1
cig2
mik1
puc1
rum1
srw1
Strain
C
Cell Length at Septation (μm)
wt
cdr2
plo1
prp19
pyp3
slm9
snf21
snf22
tif45
Strain
D
cdc10
cdc18
cdc20
cig2
mik1
puc1
srw1
5
6
7
8
9
10
11
12
13
14
15
16
17
18
19
20
Cell Length (μm)
1
2
3
4
5
6

Fig. S1. Cell length at septation for all widefield microscopy imaged strains in this study

A, Plot showing the quantification of cell length at septation for fluorescently tagged mitotic regulators. Colour indicates whether the tagged protein was included in this analysis due to its presence in our laboratory's previous haploid deletion screen (pink) (1), heterozygous deletion haploinsufficiency screen (blue) (2), or both (green). Dots indicate mean, Error bars indicate S.D. n ranges from 100 - 757 cells per strain. All strains tagged with mNeonGreen except for Cdc13 (internal sfGFP), Wee1 (N-terminal GFP) and Pka1 (C-terminal GFP). **B** - **C**, as for **A** except for genes associated with the G1 to S-phase transition (**B**) or for other genes of interest involved in mitotic control (**C**). n = **B,** 81 - 608 cells per strain and **C**, 70 - 336 cells per strain. **B** - **C**, All strains are tagged with mNeonGreen. **D**, Heatmap showing the mean cellular fluorescence intensity for asynchronous populations of strains fluorescently tagged for G1-S phase transition genes with the axis for fold-change increased to 6x from Fig 1D to prevent saturation and show the full range of fold-change for each strain.

Fig. S2.

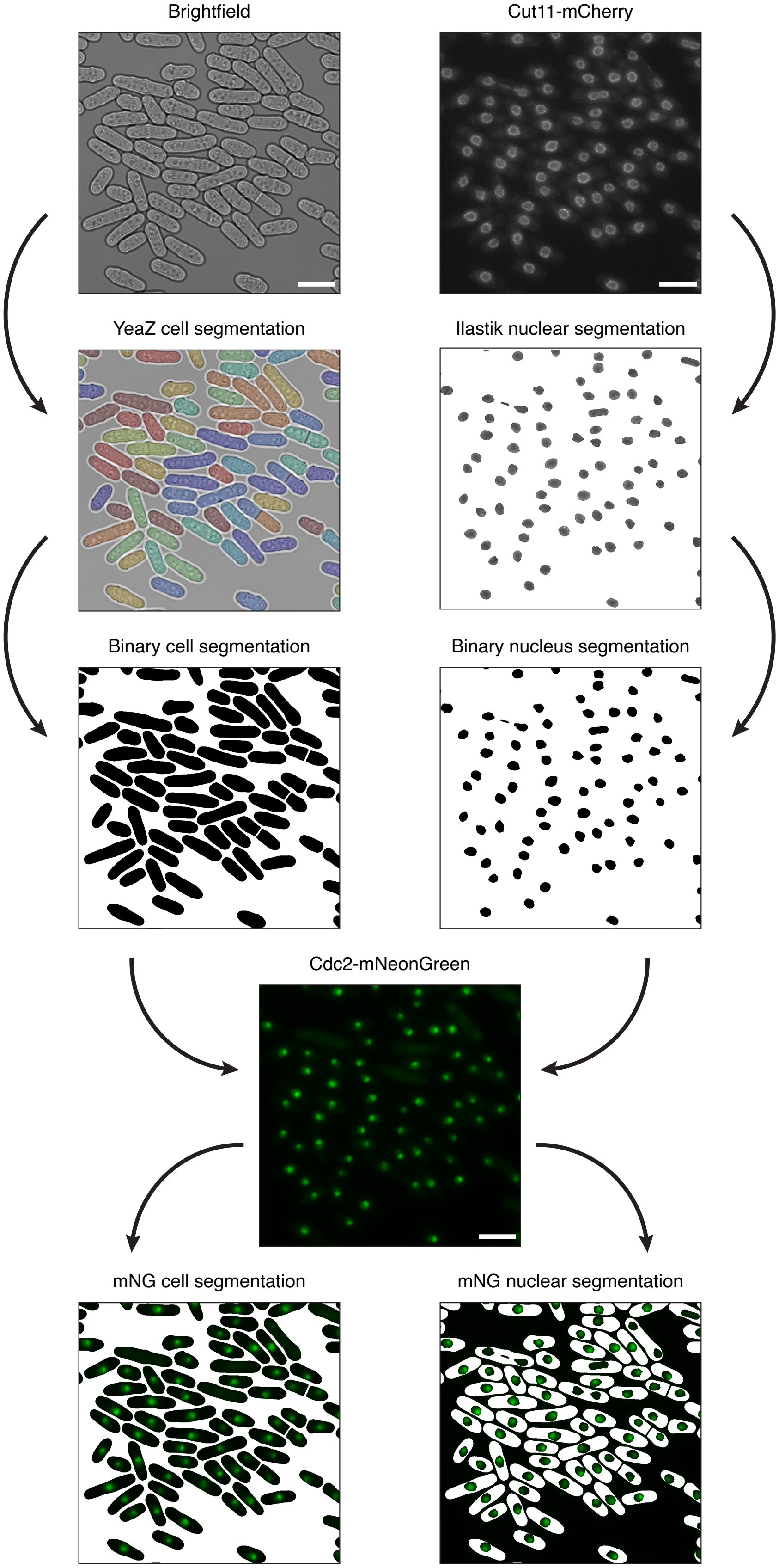
Brightfield
Cut11-mCherry
YeaZ cell segmentation
Ilastik nuclear segmentation
Binary cell segmentation
Binary nucleus segmentation
Cdc2-mNeonGreen
mNG cell segmentation
mNG nuclear segmentation

Fig. S2. Schematic of the whole cell and nuclear segmentation pipeline using *YeaZ* and *Ilastik* for widefield imaging.

Along the left, brightfield images of whole cells are segmented with *YeaZ*. Cells with septa are split into two individual cells to allow for clearer analysis of localisation and level changes occurring at the G1 to S-phase transition. Cell segmentation is exported as a binary image. Along the right, Cut11-mCh (nuclear marker) is segmented with *Ilastik* and converted to a binary image. *YeaZ* and *Ilastik* binary masks are combined and overlaid onto the tagged protein-of-interest (in this example Cdc2-mNG) to allow for whole cell measurements (bottom left), or nuclear cell-associated measurements (bottom right).

Fig. S3.

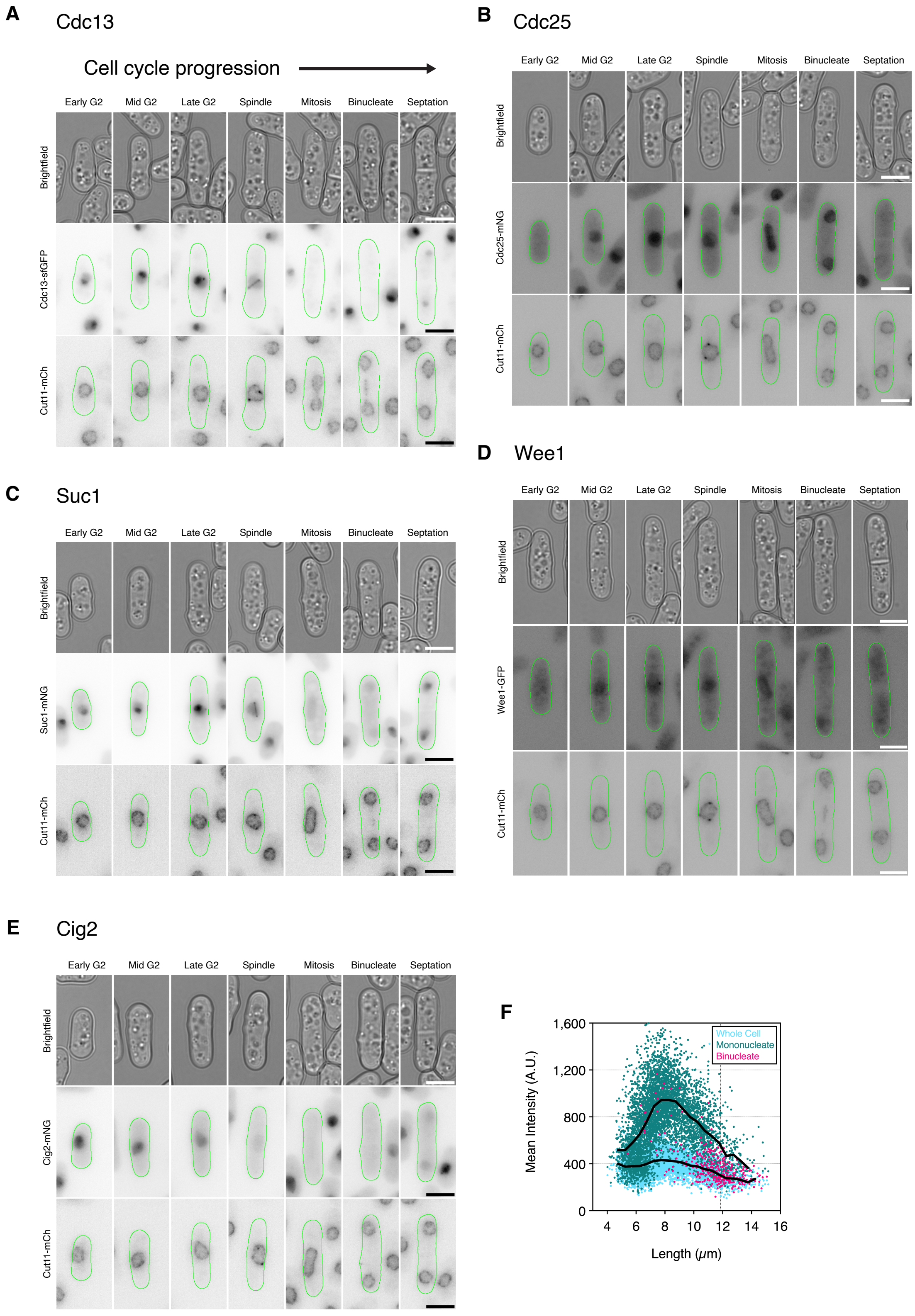

A
Cdc13
Cell cycle progression
Early G2
Mid G2
Late G2
Spindle
Mitosis
Binucleate
Septation
Brightfield
Cdc13-sfGFP
Cut11-mCh
B
Cdc25
Cdc25-mNG
C
Suc1
Suc1-mNG
D
Wee1
Wee1-GFP
E
Cig2
Cig2-mNG
F
1,600
1,200
800
400
0
4
6
8
10
12
14
16
Mean Intensity (A.U.)
Length (μm)
Whole Cell
Mononucleate
Binucleate

Fig. S3. Cell cycle montages showing nuclear accumulation of core mitotic regulators
Montages showing representative cells (green outlines), selected from asynchronous populations at progressive stages of the fission yeast cell cycle from early G2 through to septation for Cdc13-sfGFP (**A**), Cdc25-mNG (**B**), Suc1-mNG (**C**), Wee1-GFP (**D**) and Cig2-mNG (**E**). Top, Brightfield images; Middle, Inverted fluorescence images; Bottom, Inverted fluorescence images of Cut11-mCh (nuclear marker). Fluorescence images for mNG and GFP are maximum intensity projections normalised min-to-max for all pixels within each montage. Cut11-mCh images are maximum intensity projections normalised from 10,000 to 50,000-pixel values (64-bit images). Scale bars = 5 μm. **F**, Plot showing mean cellular fluorescence intensity (light blue), mean nuclear fluorescence for mononucleate cells (green) and binucleates (pink) plotted against cell length for Cig2-mNG. Black lines represent connected mean values calculated at 0.5 μm bins for whole cell values (bottom), and nuclear mononucleate values (top). Vertical dotted line indicates cell length at septation. n = 5,221 cells.

Table S1. Complete list of *S. pombe* strains used in this study

List of all the *Schizosaccharomyces pombe* strains used in this study along with their full genotypes, mating type, Nurse Lab stock number (PN…), S Curran stock number (SC…) and relevant source references and strain construction notes.

Lab Stock number	SC Stock number	Genotype	MT	Source reference / Strain construction
PN1	SC1	*972*	h-	Lab stock
PN5823	SC116	*cdc2-mNeonGreen:Kan*	h-	This study, mNG transformed into PN1
PN5883	SC310	*cdc13-sfGFP*	h-	(3)
PN5822	SC115	*cdc25-mNeonGreen:Kan*	h-	This study, *cdc25-mNeonGreen::Kan* transformed into PN1
PN5817	SC109	*cdr1-mNeonGreen:Kan*	h-	This study, *cdc25-mNeonGreen:Kan* transformed into PN1
PN5864	SC264	*clp1-mNeonGreen:Kan*	h-	This study, *clp1-mNeonGreen:Kan* transformed into PN1
PN5874	SC284	*cpc2-mNeonGreen:Kan*	h-	This study, *cpc2-mNeonGreen:Kan* transformed into PN1
PN5872	SC280	*dea2-mNeonGreen:Kan*	h-	This study, *dea2-mNeonGreen:Kan* transformed into PN1
PN5855	SC244	*git3-mNeonGreen:Kan*	h-	This study, *git3-mNeonGreen:Kan* transformed into PN1
PN5856	SC246	*git5-mNeonGreen:Kan*	h-	This study, *git5-mNeonGreen:Kan* transformed into PN1
PN5861	SC258	*gpa2-mNeonGreen:Kan*	h-	This study, *gpa2-mNeonGreen:Kan* transformed into PN1
PN5867	SC270	*nif1-mNeonGreen:Kan*	h-	This study, *nif1-mNeonGreen:Kan* transformed into PN1
PN5868	SC272	*nud3-mNeonGreen:Kan*	h-	This study, *nud3-mNeonGreen:Kan* transformed into PN1
PN5859	SC252	*nup45-mNeonGreen:Kan*	h-	This study, *nup45-mNeonGreen:Kan* transformed into PN1
PN5853	SC240	*nup97-mNeonGreen:Kan*	h-	This study, *nup97-mNeonGreen:Kan* transformed into PN1
PN5882	SC300	*nup184-mNeonGreen:Kan*	h-	This study, *nup184-mNeonGreen* transformed into PN1
PN5854	SC242	*nup189-mNeonGreen:Kan*	h-	This study, *pab2-mNeonGreen:Kan* transformed into PN1
PN5871	SC278	*pab2-mNeonGreen:Kan*	h-	This study, *pab2-mNeonGreen:Kan* transformed into PN1
PN5812	SC69	*pka1-GFP:Hph*	h-	This study, *pka1-GFP:Hph* transformed into PN1
PN5866	SC268	*pom1-mNeonGreen:Kan*	h-	This study, *pom1-mNeonGreen:Kan* transformed into PN1
PN5862	SC260	*ppa2-mNeonGreen:Kan*	h-	This study, *ppa2-mNeonGreen:Kan* transformed into PN1
PN5863	SC262	*pyp1-mNeonGreen:Kan*	h-	This study, *pyp1-mNeonGreen:Kan* transformed into PN1
PN5873	SC282	*sal3-mNeonGreen:Kan*	h-	This study, *sal3-mNeonGreen:Kan* transformed into PN1
PN5850	SC234	*sgf73-mNeonGreen:Kan*	h-	This study, *sgf73-mNeonGreen:Kan* transformed into PN1
PN5851	SC236	*ski3-mNeonGreen:Kan*	h-	This study, *ski3-mNeonGreen:Kan* transformed into PN1
PN5869	SC274	*snf5-mNeonGreen:Kan*	h-	This study, *snf5-mNeonGreen:Kan* transformed into PN1
PN5870	SC276	*sol1-mNeonGreen:Kan*	h-	This study, *sol1-mNeonGreen:Kan* transformed into PN1
PN5865	SC266	*SPAC27E2.03c-mNeonGreen:Kan*	h-	This study, *SPAC27E2.03c-mNeonGreen:Kan* transformed into PN1
PN5820	SC112	*suc1-mNeonGreen:Kan*	h-	This study, *suc1-mNeonGreen:Kan* transformed into PN1
PN5841	ER28	*lys1+::wee1-GFP_wee1_wee1Δ:ura4+_ura4-D18*	h-	(4)
PN5867	SC248	*zfs1-mNeonGreen:Kan*	h-	This study, *zfs1-mNeonGreen:Kan* transformed into PN1
PN5813	SC95	*cdc10-mNeonGreen:Kan*	h-	This study, *cdc10-mNeonGreen:Kan* transformed into PN1
PN5860	SC254	*cdc18-mNeonGreen:Kan*	h-	This study, *cdc18-mNeonGreen:Kan* transformed into PN1
PN5826	SC119	*cdc20-mNeonGreen:Kan*	h-	This study, *cdc20-mNeonGreen:Kan* transformed into PN1
PN5818	SC110	*cig2-mNeonGreen:Kan*	h-	This study, *cig2-mNeonGreen:Kan* transformed into PN1
PN5825	SC118	*mik1-mNeonGreen:Kan*	h-	This study, *mik1-mNeonGreen:Kan* transformed into PN1
PN5815	SC99	*puc1-mNeonGreen:Kan*	h-	This study, *puc1-mNeonGreen:Kan* transformed into PN1
PN5819	SC111	*srw1-mNeonGreen:Kan*	h-	This study, *srw1-mNeonGreen:Kan* transformed into PN1
PN5878	SC292	*cdr2-mNeonGreen:Kan*	h-	This study, *cdr2-mNeonGreen:Kan* transformed into PN1
PN5877	SC290	*plo1-mNeonGreen:Kan*	h-	This study, *plo1-mNeonGreen:Kan* transformed into PN1
PN5879	SC294	*prp19-mNeonGreen:Kan*	h-	This study, *prp19-mNeonGreen:Kan* transformed into PN1
PN5816	SC108	*pyp3-mNeonGreen:Kan*	h-	This study, *pyp3-mNeonGreen:Kan* transformed into PN1
PN5875	SC286	*slm9-mNeonGreen:Kan*	h-	This study, *slm9-mNeonGreen:Kan* transformed into PN1
PN5880	SC296	*snf21-mNeonGreen:Kan*	h-	This study, *snf21-mNeonGreen:Kan* transformed into PN1
PN5881	SC298	*snf22-mNeonGreen:Kan*	h-	This study, *snf22-mNeonGreen:Kan* transformed into PN1
PN5876	SC288	*tif45-mNeonGreen:Kan*	h-	This study, *tif45-mNeonGreen:Kan* transformed into PN1
PN5814	SC96	*cig1-mNeonGreen:Kan*	h-	This study, *cig1-mNeonGreen:Kan* transformed into PN1
PN5821	SC114	*rum1-mNeonGreen:Kan*	h-	This study, *rum1-mNeonGreen:Kan* transformed into PN1
PN5845	SC347	*cut11-mCherry:Nat*	h-	Lab stock
PN5886	SC313	*cdc2-mNeonGreen:Kan_cut11-mCherry:Nat*	h-	This study, SC116 x SC348 (*cut11-mCherry:Nat* h+)
PN5899	SC318	*cdc13-sfGFP_cut11-mCherry:Nat*	h-	This study, SC310 x SC348 (*cut11-mCherry:Nat* h+)
PN5888	SC317	*cdc25-mNeonGreen:Kan_cut11-mCherry:Nat*	h-	This study, SC115 x SC348 (*cut11-mCherry:Nat* h+)
PN5895	SC334	*suc1-mNeonGreen:Kan_cut11-mCherry:Nat*	h-	This study, SC112 x SC348 (*cut11-mCherry:Nat* h+)
PN5890	SC319	*lys1+::wee1-GFP-wee1_wee1Δ:ura4+_cut11-mCherry:Nat_ura4-D18*	h-	This study, ER28 x HC121 (*cut11-mCherry::Nat ura4-D18 leu1-32 ade-* h+)
PN5899	SC342	*cig2-mNeonGreen:Kan_cut11-mCherry:Nat*	h-	This study, SC110 x SC348 (*cut11-mCherry:Nat* h+)
PN5896	SC335	*cdc18-mNeonGreen:Kan_cut11-mCherry:Nat*	h-	This study, SC254 x HC121 (*cut11-mCherry::Nat ura4-D18 leu1-32 ade-* h+)
PN5887	SC315	*mik1-mNeonGreen:Kan_cut11-mCherry:Nat*	h-	This study, SC118 x HC121 (*cut11-mCherry::Nat ura4-D18 leu1-32 ade-* h+)
PN5885	SC312	*cig1-mNeonGreen:Kan_cut11-mCherry:Nat*	h-	This study, SC96 x HC121 (*cut11-mCherry::Nat ura4-D18 leu1-32 ade-* h+)
N/A	SC333	*rum1-mNeonGreen:Kan_cut11-mCherry:Nat*	h-	This study, SC114 x HC121 (*cut11-mCherry::Nat ura4-D18 leu1-32 ade-* h+)

Table S2. Primers used for C-terminal tagging of proteins analysed in this study

List of all *Schizosaccharomyces pombe* genes fluorescently tagged in this study, with tagging primers for pFA6A C-terminal tagging of full-length proteins and checking primers.

Gene	Pombase ID	Screen	Tag	Fw Tagging Primer (5'-3')	Rv Tagging Primer (5'-3')	Fw Checking Primer (5'-3')	Rv Checking Primer (5'-3')	Reference
cdc2	SPBC11B10.09	HI	mNG	AGGCTATGCTTGTTTATGACCCTGCCCATCGCATTAGTGCAAAACGAGCTTTGCAACAAAATTATCTTCGTGATTTTCATGATTCTGCTGGATCAGCTGGC	AACTGATATCAAGAAACACAGCAAAGTACAGATAAAGTCAAGGATAGCGTTTTTAAAGGTTTAATAATAAGAGACGAAAAGAATTCGAGCTCGTTTAAAC	CGTTGGATGTATTTTTGCTGAA	TATGTTTTGAACAAACGCCAAG	
cdc13	SPBC582.03	HI	sfGFP	N/A	N/A	N/A	N/A	(3)
cdc25	SPAC24H6.05	HI	mNG	TTGGCCAAAGTGTGTTAGCTTCCCCAGACGTTAATGATTCTCCTACTGCCATGCATTCCCTCTCTACACTTAGAAGATTTGATTCTGCTGGATCAGCTGGC	AGAAAAAACTTAGGTTTAGAAAGTTGAATATATAAGAGTATACTTCAGGCTAGGTAAAGTATTGAGTCAGCCTAAAATCAGAATTCGAGCTCGTTTAAAC	AAACCGTTGTGACCCAATTAAC	GAGCAATTAGAATGGACTTCGG	
cdr1	SPAC644.06c	HI	mNG	CTACTAATGCAAGATATACTCCCAGAAAAGTTTCTTCCGGTTCTGTATTACGAAAGATTTCTTCATTCTTTCGGAAGGATGATTCTGCTGGATCAGCTGGC	ACATTTGAATAGCCGCCCACAGTATAGACATTAATGGTTGATTTTGAGGAAGAAGACTGTGTTTTTGAAAAAATTCCTATGAATTCGAGCTCGTTTAAAC	GCATCTTTCCTAGCACCACTCT	GTGTGCCCAAATCATTAACAGA	
clp1	SPAC1782.09c	Hap	mNG	GTGTTAGCATGTCATCACTTAACAATACTTCTAATGGCCGTGTTGCTAAACCTAAGCCTTCTAAAAGCCGGCTAATTTCTGATTCTGCTGGATCAGCTGGC	GACACAGTATAATTCAAAGTTAGTTTATATGAAAAAAGGAATGTAAAAACTGCCATTTAAACCAGTAATTACAGGTTTAGAATTCGAGCTCGTTTAAAC	ATCCAAAATGAGAACAAAGCGT	GCGCTAAATCAGGGAATATTTG	
cpc2	SPAC6B12.15	HI	mNG	CTCTTACCTGGTCTCCTGATGGCCAAACTTTGTTCTCTGGCTGGACTGATAATCTCATTCGTGTCTGGCAAGTTACCAAGGATTCTGCTGGATCAGCTGGC	ATTCAGAGTTTAAAGAAAACGATGCACCCTAAAGCCATTCATCATTATCGTCTTATGGGACAACAATTAAAATCTTATTTGAATTCGAGCTCGTTTAAAC	TACCTTGATGCTTTGGGATCTT	TGGTGTTTAAAGGGATTTTTGC	
dea2	SPBC1198.02	HI	mNG	CTGTTCAAAAATGTGTTAAGGAGTATACTGCTGAAATTCAACAACCCAAAACCCTTGAAACAGCTGTGGAAGTTCAAGCTGATTCTGCTGGATCAGCTGGC	AAATGAAGATAGTCTAAAGAATATTTTAAAGAATGTAAAAAGGAAAGAATGAAAACAAATACTCAATCACCGACAATTGGAATTCGAGCTCGTTTAAAC	GGGTTTTTATTGCTAATGCTGC	TGCGTTTTTCGATAACAATGAC	
git3	SPCC1753.02c	Hap	mNG	GAGATTCGAGTAAAAGTTATTGGGATCAAATAAAGGAGTTGACTTTAAAATGGTGGCGGGGTAAATTTGGTGAGGAAAAAGATTCTGCTGGATCAGCTGGC	CGTCATAAAAAGAAATTATAATACAAGAAGAGGAGCTTGTGAGACTTCACCATAAACAAAAATAAAAAGAACAAATCCTTGAATTCGAGCTCGTTTAAAC	AATGATATGCAAGATGATCCCC	CATTCACTGGATGGCTTTAACA	
git5	SPBC32H8.07	Hap	mNG	CTTTAGCTTTAACTTCTGATGGAACAATGTTGGCAACTGGCTCTTGGGACGAATGTGTTCGTCTCTGGTCTTCGTCAGGGGATTCTGCTGGATCAGCTGGC	GTAAATGTATAAGAAAAAAAAACAAAACAAACATCATCATTTCCAAAGACAAAAAAAAAAGGAGAAAAAAAATCATTAATGAATTCGAGCTCGTTTAAAC	CCCGGAAATACATCGGATATTA	AGCAGTCAACCTCCTAGAATCG	
gpa2	SPAC23H3.13c	Hap	mNG	ACACATCTAACATAAAGGTTGTCTTTTCTGCCGTTAAGGAAACAATTCTACAACACAGTCTGAAAGAAGCGGGAATGTTTGATTCTGCTGGATCAGCTGGC	CTCTAGACATATGCAGACAGTAAGAGGTCTTCTGTTAACATATGAATAATGGTAGGGTAAATTTTATCTTCAAATTAAAAGAATTCGAGCTCGTTTAAAC	ATTTGTTTCGGAAGAAACTGGA	CAGACAAATCGGTGATTTCAGA	
nif1	SPBC23G7.04c	Hap	mNG	ATTTAGAATCTCACTCACTGAAGTTTTCTACAAAGCCAAAAGCTAAATTACGAAGTTTGATTACTTCTGTAAGGTATTTGGATTCTGCTGGATCAGCTGGC	ATTGTGGAAAATGCAAATAAATGAATAGGACAAAAACAAAAAAAAAAGACAAAAAAAATGGTAAAAGAATGAGAGGGATTGAATTCGAGCTCGTTTAAAC	ATCGTTATCTGGCTTTGCATTT	TCGTCGGAATCACTTTTTATCC	
nud3	SPBC19F8.02	Hap	mNG	AACAAAAACGAAAAGATGTACTGCAAAATTTCATGAAACAACATCCTGAACTAGATTTTTCAAATGTTAGAGACCAGATTGATTCTGCTGGATCAGCTGGC	AAATTTCTACATTTTTATCAATAGAGCTTAGAAAGTTTTGTAACAATTGTAATTATACATATATAAAATCATGAAACTATGAATTCGAGCTCGTTTAAAC	CTGTCGAAGAACAAGAAAGGCT	AGAATGCCGATTTGCTGTTTAT	
nup45	SPAC22G7.09c	HI	mNG	TTAGTAATAGATTTGCTCAAGTTCACGATGAAGTAAAGCGTCTACAAGTGAACACTTCAACGTCCTTGCCCTTTATAAGTGATTCTGCTGGATCAGCTGGC	AATTTGTCTACTTTAGAGATTTTTATCTATTTATTTTTTTATGATGTTAAGGATTATGAAGTCCCTTTAATGATCGCTAGAATTCGAGCTCGTTTAAAC	TATGCTGCCACTATTGGTGAAC	AGCACTACATGGATGCAACAAG	
nup97	SPCC290.03c	HI	mNG	TCATGTACAGTTTCCTGATAGAATATCGTATGCCTTCTCAAATTCTTGAACAGTTAAATCGCTGCGAAATAGAAATGACAGATTCTGCTGGATCAGCTGGC	AATTAGGACTCAATGAATGTATAGGCATAAAAATTTAGTGAAGTCAAGTAAACATAGAATAAACAACCTTAAAAAATAAGAATTCGAGCTCGTTTAAAC	AATTTTACCCCTTGACCCACTT	AGAGAAACAGCCTCAGGAATTG	

Gene	Pombase ID	Screen	Tag	Fw Tagging Primer (5'-3')	Rv Tagging Primer (5'-3')	Fw Checking Primer (5'-3')	Rv Checking Primer (5'-3')	Reference
nup184	SPAP27G11.10c	HI	mNG	TGCAACAATTTAATCCCGCATTACTACAAGAAATTAGACTTGCTGAATTGAAGATTGAGATGCTGGAGGCATCGACTATTGATTCTGCTGGATCAGCTGGC	GTAGAAATTTTGCTGCATTGTGAAAATGCAGTAAATATCTTTTTAAAGAATGACTTCAGCAAATATTATCAAAACTACATGAATTCGAGCTCGTTTAAAC	GGTGCTGAACAAATCGTTATGA	GAAATTTTGCTGCATTGTGAAA	
nup189	SPAC1486.05	HI	mNG	CACTTACGGATGCTATATGTAATCTCCCCTTACCACTTGCTGACAGTCTAGCGAATTTACAAAATATATCTGTGCAATTTGATTCTGCTGGATCAGCTGGC	AACTCAGAACATATATTCCGATTTCATTAAATATAGATTTTTTTTCTAATTCATCGTATAAACCAATCTAGAATACTAAAGAATTCGAGCTCGTTTAAAC	TTAACGCGCTTTGTAATGAAGA	TTATGAATGATTGCGCGTTTAG	
pab2	SPBC16E9.12c	Hap	mNG	GTCGTGGTCGCGGACGTGGACGTGGACGGGGTCGTGGCAGAGGAGGATATCGTGGAAGAGCCCGTGGTTTCGCTCCGTATGATTCTGCTGGATCAGCTGGC	TTTCCCACCTAACTGTTATTGGTGACGATTTGAATGCTCATAATTCATTTTTCACTTTTTCAAGTCATCAAAGCGATTACGAATTCGAGCTCGTTTAAAC	GATGTTGCATGAACGACCTTTA	GCTGGCATTGTAAACACAAAAA	
pka1	SPBC106.10	Hap	GFP	AATTTGACGCTTATGCTGATGTAGCTACGGATTATGGAACATCTGAAGATCCTGAATTTACTTCTATCTTTAAGGACTTTCGGATCCCCGGGTTAATTAA	GCTCAACGCTTTAAGGCAATAGTACAATGAATCAATAAGCATTCGAGTATCTTAAAACAAAACAAACGTGGCACAAAACAGAATTCGAGCTCGTTTAAAC	ATATCCTTGAAGGCAAGGTCAA	TGAAGAAGAAATGACTTGGGGT	
pom1	SPAC2F7.03c	HI & Hap	mNG	CCAATCTTTTGACAAATTTAGATTATTCAATAATTTCAGATAATGGTTTTTTACGAAAGCCGGTAGAGAAATCCCGGCCGGATTCTGCTGGATCAGCTGGC	GTCAAATTAAAAAGAAAAAAAAGTTGATGTACTTGAAACGCAAGCAAAAGGTTTTCAACCTATTCCAAGGTAATGTGTGAGAATTCGAGCTCGTTTAAAC	AGAGCCCTCTAACCAAGCTTCT	AACTAGCATGGTTGACACGTTG	
ppa2	SPBC16H5.07c	HI & Hap	mNG	ATCAAGTCTTTTTACAATTCGATCCTGCCCCACGAGAAGGCGAACCGGTAATAGCTCGAAGGACACCAGACTACTTCCTTGATTCTGCTGGATCAGCTGGC	TTAACTCAAAAATCAGAAAGGTGGATAAATTTTTTGAAACAATCACAATCGATTGATCACTCATTAGAGAATTGAAACTTGAATTCGAGCTCGTTTAAAC	CCCATCAGCTAGTTATGGAAGG	CAATGGCTTGAAGTGGAATACA	
pyp1	SPAC26F1.10c	Hap	mNG	AAACCTTCACACAATTTAAATATGTGTATGACTTGATCGATTCTTTGCAAAAATCTCAAGTTCATTTCCCGGTTTTAACAGATTCTGCTGGATCAGCTGGC	AGACACTTTACAAGTACAAGAAATAAAGGAATCGATTAAAACACGAATATATATTGCCAAGAAAAATCCAGTCAAAAATTGAATTCGAGCTCGTTTAAAC	GATTCTTCAGACGTCGTTTTCC	ACGACCACTTCGCTTAAACTCT	
sal3	SPCC1840.03	HI	mNG	CTCGTTTCCCTGCTGATCAAGTTAATTCTGTCATTGCCACTTTGAGTGTCGATAATCAGAGAGCTTTGTCTGCACATTTTGATTCTGCTGGATCAGCTGGC	CCTAACCTAACCTAAACTAACTAAAGCATTTTGAGGAACCAAAGAAAATATTGAAAGACTTAGAGTGAAAGAAATGAAATGAATTCGAGCTCGTTTAAAC	CAAATGCCAACGATTATTACGA	GCAATAAGTGAAAACCTAGCCG	
sgf73	SPCC126.04c	Hap	mNG	TTCGACATGGTGAGCAGGGTATGCAAGTTACTGGTACTATATTGGGACGAGTGATTCCTTTTAGTGCCCGCCAACCACTAGATTCTGCTGGATCAGCTGGC	TGGTCAAGTAGACCAAAATGTAAACTATAATCTCATAAAGAATTTAAATCATCAACCGAAGCAAAATCAAAAGAATTTCTGAATTCGAGCTCGTTTAAAC	TTGCAGCTTGTCAAAAGAAAAA	GGGCTAAAGGATTGATTGTGAG	
ski3	SPCC1919.05	Hap	mNG	ACTTGGTTCCATGGGATTCCGCAAATTGGAAAGCTTTGCATGGTGTTACTCATGAGGCACTAGTTTCAAGTGATGCTTCGGATTCTGCTGGATCAGCTGGC	ATTTACAGGTTCCACAAACATTTCCTAACTTGGTAGATATAGTTCGCTGGGGTCATTAAGTTGATAATAAATACAAGTTCGAATTCGAGCTCGTTTAAAC	TGCTCATTTGAAGAAAGACGAA	AAAAATGGCTCTAGGTAAGGGG	
snf5	SPAC27F7.08c	Hap	mNG	ATTTCCATATCCACTTGTTGTCAAGCTCGCATCAACAAAAAAAGGAGGTGAGAATGAATACGGTTTTGGATAGAAATACTGATTCTGCTGGATCAGCTGGC	AGAAACTGGTCCGTCGAGCTATCCATAACGTGAGTCAAATCTTGATTATGGTCTTTGATAAAGATTCTTCCAAAATTGTAGAATTCGAGCTCGTTTAAAC	CTGCTGAAAGGATGACTGTGAG	CGAGAAACAAGGAAAGACTGCT	
sol1	SPBC30B4.04c	Hap	mNG	TAAAAGAGATCTCAAATTTATTGGATCGAACTGGGGACAGTGATGCGTCTTTAGAAAATACGGATGATAAATCTGGCATTGATTCTGCTGGATCAGCTGGC	AGACAATATACATACAGAAACGCATCAGATTAACTAGAATGAAGACAAAAATATTAAACGTTTGGAAAATTATCTACAAAGAATTCGAGCTCGTTTAAAC	ATATCTACGGTGCGTCGACTTT	AATTGTTGCAATGCGTAGAATG	
SPAC27E2.03c	SPAC27E2.03c	Hap	mNG	CTGCCGGTAAGTACCTTACCAAAGGAAAAGAATATGTTATGGAGAGTGGTGACATTGCTCATTGGAAGGCTGGCAAGCGAGATTCTGCTGGATCAGCTGGC	TAATACAATTTGCACTTTATAATATACTCTTGTTCAAATATTTTAAATTCAAAAAAAAAAAAAGCAATAGTAAGCATAATGAATTCGAGCTCGTTTAAAC	AAGTTCGCTCTTGGACTATTCG	ATATTGGCGGGGTTCTTTTAAT	
suc1	SPBC1734.14c	HI	mNG	TCCATGTCCCAGAGCCACACATCCTGCTATTTAAGCGTGAAAAAGATTATCAAATGAAATTTAGTCAACAACGGGGTGGTGATTCTGCTGGATCAGCTGGC	CCTAAAAAGTCAATATGTTTCGCATCCATGAACTAATATTTTTTTGATAAAGTAAAACAGAAACGTTGGAGTTGACAAACGAATTCGAGCTCGTTTAAAC	TTTGTTTTCCCCTAAAACGAGA	AGCAAACATAAATGGCGAAGAT	
wee1	SPCC18B5.03	HI	GFP	N/A	N/A	N/A	N/A	(4)

Gene	Pombase ID	Screen	Tag	Fw Tagging Primer (5'-3')	Rv Tagging Primer (5'-3')	Fw Checking Primer (5'-3')	Rv Checking Primer (5'-3')	Reference
zfs1	SPBC1718.07c	Hap	mNG	ATTGTCCTTACGGATTGCGATGCTGCTTTTTGCATGATGAATCCAATGCTCAAAAAAGTGCAACTATTAAGCAATCTCCTGATTCTGCTGGATCAGCTGGC	TAAGCGTCATAATAAATTAATCTCACCCAGATTGCATAACAACAAAGAACAAAAGTCATCTTATACAAGCTGATGTGATAGAATTCGAGCTCGTTTAAAC	CTGGCAGTAGTAATGGGGTAGC	ATGACTGGCAAACTCAAACCTT	
cdc10	SPBC336.12c		mNG	TGGAAAGTGACGGTCAACAGGGAGAAGTAGATATGGGTCGAGTTGCTGGATTCTTACGTGTTGTTAAAGAACATCAAGCAGATTCTGCTGGATCAGCTGGC	GGTAGGATTCTATATTAAGAAAAAAATTAAAGTAGTTAATTATTTCAGGACACTTTAGTGGTAAACCACAAAAAGCAATAGAATTCGAGCTCGTTTAAAC	GAATTAGCGAACAAACTTTGGC	GTTAATGTCGTAGAAGCCGAGC	
cdc18	SPBC14C8.07c		mNG	TTAGTTTGCTTGTTCCAGAAATGGATGTCATTACAGCTGTTGGAGACATTGGTACCTTAAAACGATTTTTTGACAGAAGAGATTCTGCTGGATCAGCTGGC	TTCGGCAAAATTTGAAAACCGTAACGCGTGAAATAAAAATATAATGAATAGAAAAAAAGAATGAGAAAGAAATGATAGTAGAATTCGAGCTCGTTTAAAC	GAGTGTTGCCGATGTATTTGAA	GAGACCCCAAAATTGAGAAGTG	
cdc20	SPBC25H2.13c		mNG	ACGTTTATCAATCTGTTGCTGATTTTTACGAGTTTTCTATATTGCAAAATTCTGTTCAGTCCATACTTTCTGTGCTGAACGATTCTGCTGGATCAGCTGGC	GCAGATAAAATCAAAATAACTTATAAAATACATATAAAGACAAAAAAGCCAAACTCATGCTCATGGTCAGGCGAGAAATGGAATTCGAGCTCGTTTAAAC	ATGGTCATTAAAGGAACGCTGT	AGAGCATGAGTGGAAAAATGGT	
cig1	SPCC4E9.02		mNG	ATGCCATTTACCGAAAGTACTCGGAGAATAGAATGAAGCGAGTTAGCGCCTTTGCTCACAACTGGGTACTAAGTGTGATTGATTCTGCTGGATCAGCTGGC	ATCCGTAATAATAAATATCGTAGGCTTAATAATAGCAAACTAACTCAGAATATCATTGTTAACAACTTCTGAAAGCAAACGAATTCGAGCTCGTTTAAAC	TTGTCTATGCAGATGTTGGGTC	CAGTAGTTCCAAGTCTCTGGGG	
cig2	SPAPB2B4.03		mNG	GTCAACGATACTCAGTCAATCGTACGGACGATGATGACCTTCAATCAGAACCGTCTTCTTCTTTAACAAATGATGGTCACGATTCTGCTGGATCAGCTGGC	TATGATAATAAATAATAAAAGAAGAGCTCAACCTCATATTCAACAGAATATTTGCGTAAGAAAATTTATAAGAGCGTTCGGAATTCGAGCTCGTTTAAAC	AAAGCTCTGGTGGGTATGAAGA	TTATCAAGGAGGAAAGGCTTGA	
mik1	SPBC660.14		mNG	TGCCGGAAATGATATTCATCTCTGAGCATTCTCAAAAAGCTGCAATTATCTACGAAGACCATAACAGTTGGTTAGAAACTGATTCTGCTGGATCAGCTGGC	AAATACAAATTAATGAACCATGGAAGAACGCAAAATTCATTCCCTCATTGTGGGGCTAAAATATTCGCACACAGAATCCCGAATTCGAGCTCGTTTAAAC	GGTGAGGTGTGCTGAATCATTA	ATTGGGGTAAACACAAGGTCAC	
puc1	SPBC19F5.01c		mNG	TTTTAGCAAAGAAATATCCGGAACAATGCGCAATGGCTGCCTGGTGCAACATGACTGAAAAGGATACTGAGCGTACTTTGGATTCTGCTGGATCAGCTGGC	ATTTTGAAATTATATCTCCATTACATGTTTGTCTAGAAGCATTGCAATATATATAAATCGAAGAAGAAGCAATGTTAAAGGAATTCGAGCTCGTTTAAA	ATCGTTTCTTTTGACACACCCT	ATATGAGCATAGTTGCAAACCG	
rum1	SPBC32F.09		mNG	AAATGCGTTTACCTGCGTATTCATCACCACAAAAATCACGATCTAATACAAAAGATGAAAACAGGCACAATTTATTACGAGATTCTGCTGGATCAGCTGGC	ATGAATAAGGCAGAAGAGTATTTCGTGATTGGGCATTTATATAAACGGTATCAAACACAATTACAAAATGCGAAAAAAAGGAATTCGAGCTCGTTTAAAC	TTCAGGCCTGGAACTGATTTAT	ATGCCGTAAAAGGTTGCTAAAA	
srw1	SPAC144.13c		mNG	TCTGGAAGCTGTTTGATTCTAAATCAAAACACTCCGCTTCCACTATGAGTTCTCCATTTGACCCTACAATGAAAATAGATTCTGCTGGATCAGCTGGC	AAAGCGACAAAGGTGTAATATTTTAGTAATAAGATGACAAATATAATGTACAAAAGACTTCAAAGAGTGAATGTGTTGCAGAATTCGAGCTCGTTTAAAC	TCTGGCTTCTGGAGGAGGTA	CTGTCACATTGCCAACAGAAA	
cdr2	SPAC57A10.02		mNG	CGGCATCCAGACCTGTTTCTCGAATGAGTGTAAGTAGTAGTCCTTTTGCTGTATTTCGTCAACGACAATCCGTCCAAAGTGATTCTGCTGGATCAGCTGGC	CCAAAGCATCACGAGAAAAATGAAGTTTGCAAAGGTTTTGGAGAATCAAAAAAAAATGATAATAATAATAATAAAAGAATGAATTCGAGCTCGTTTAAAC	CTGTGAGTGCTTCGATATCTGC	ACATTGTAGCAGAGCAGCAAAA	
plo1	SP23C11.16		mNG	CATTTTCAGAAGACTTGAGATCTCGCTTAAAGTATATTCGCGAGACGTTGGAATCGTGGGCGTCGAAAATGGAAGTGAGTGATTCTGCTGGATCAGCTGG	TAGTACAAAAATGAGGATGTGAAATATGGAAAAATAGACAGCATAGTAACTTAACGCCCAAGTATAGATTAACGGTATTAGAATTCGAGCTCGTTTAAAC	CGCACGGAAGATCATTGTATTA	GTCGCCTTAAGACAGAATTTGC	
prp19	SPAC29A4.08c		mNG	CTATTTCTAATTTGGTTTGGTTAAATGAGTTACACCAGTTGTTGTTTAGTACTTCAAATGGAGCCATTCTCCGGTTGGGTGATTCTGCTGGATCAGCTGGC	ACTTAAGTTAAAGAGCCAAAATCTTACTAAATACGAAAAGCCTAATGGAGGAGCACATAATTAAAGGATTAAAAATTGGGGAATTCGAGCTCGTTTAAAC	ATTTGGTGAAAACGGTTATTGG	CGAGCCTGATCTTGAGAAAGTT	
pyp3	SPAC11E3.09		mNG	AATCGGTCGATCAGCTTGTTTTTCTTTATACAGTATCTCAAGAGCTGCTTCAAGGGAAGGAATTTCTTCTTCCTCAGTTAGATTCTGCTGGATCAGCTGGC	AATAATATAATGTTAATTCGAAAATTCATGAGACGAGTTTTTAACATCGAATCGACTATCAGCAAACTCTAAACCAAAGGGAATTCGAGCTCGTTTAAAC	TAGTTTCCAGTCTTCGGTCACA	TGAATCACAAAGGATGAACTGC	
slm9	SPBC15D4.03		mNG	TAATTCATACAGCAAAATACCGAGATATGCAACGAATTACTTCTCAATATTCTGACTTATTACGACGATCTGCACTTTTAGATTCTGCTGGATCAGCTGGC	GAGATGTTTTGATATATTATGTTTACGTTTTAATTTAAATTAGTTATACAATCTGTTCGTAGCAGAAGTAGTTGCAGCAAGAATTCGAGCTCGTTTAAAC	CAAGGTAGGAAATCGACTTTGG	TATGCGGTAGATGTCAACGAAC	

Gene	Pombase ID	Screen	Tag	Fw Tagging Primer (5'-3')	Rv Tagging Primer (5'-3')	Fw Checking Primer (5'-3')	Rv Checking Primer (5'-3')	Reference
snf21	SPAC1250.01		mNG	AGGATGGCACATTAGCAACGCTTCGCGGAATGGAGGCGGAGGCTACATCGCAATTGGAAGACAGAATTGAAAATGAGGCTGATTCTGCTGGATCAGCTGGC	AAAAAAACAAAAAAAAGACAATCTGTGTTTTTATGAGACTTCAGCACTTGCATCTATAGTAGTTCAAAAAAAGCAATTAAGAATTCGAGCTCGTTTAAAC	CGCACTTGATGCAATTAGAAAG	ATCATCTGAGCTAGTGTTGCCA	
snf22	SPCC1620.14c		mNG	TACCGTTGGATTCTGGTATAGTAAGCGCCGAAGATGACAAAGTTATTACTTATGAAGATTCTTCTTCTTCTTATTCGGAGGATTCTGCTGGATCAGCTGGC	ATAGTCCTCACCTAACAAAATGTACCAAAATATTATAAACAAGGCATTAAAATAAAACGACAAAAGGTAAAACGTTAGTCGAATTCGAGCTCGTTTAAAC	CAATTGTTTACGAGGATGCAAA	AGGCAGCTCTACATACGACTCC	
tif45	SPAC16E8.15		mNG	GATCGGAAACTATAGAATTTAGCGCTCATGAAGATTCTTCCAAGTCTGGTAGCACTCGCGCCAAAACTCGCATGAGTGTTGATTCTGCTGGATCAGCTGGC	CAATTATAAGCAAATTAGCTATGCAGCATGGACCTTTTAAACTCCAAAGTTATTTTGTAAACAAAGAGCGTTTTTATCATGAATTCGAGCTCGTTTAAAC	TGCGTAAAGGATTTTACCGTCT	TTCGAAAATTATCCACCCATTC	

SI References

1. F. J. Navarro, P. Nurse, A systematic screen reveals new elements acting at the G2/M cell cycle control. *Genome Biol.* **13**, R36 (2012).
2. N. Moris, *et al.*, A genome-wide screen to identify genes controlling the rate of entry into mitosis in fission yeast. *Cell Cycle* **15**, 3121–3130 (2016).
3. J. Kamenz, T. Mihaljev, A. Kubis, S. Legewie, S. Hauf, Robust Ordering of Anaphase Events by Adaptive Thresholds and Competing Degradation Pathways. *Mol. Cell* **60**, 446–459 (2015).
4. H. Masuda, C. S. Fong, C. Ohtsuki, T. Haraguchi, Y. Hiraoka, Spatiotemporal regulations of Wee1 at the G2/M transition. *Mol. Biol. Cell* **22**, 555–569 (2011).
